# What characterizes the work culture at a hospital unit that successfully implements change – a correlation study

**DOI:** 10.1186/s12913-017-2436-4

**Published:** 2017-07-14

**Authors:** Beate André, Endre Sjøvold

**Affiliations:** 1 Department of Public Health and Nursing, Norwegian University of Science and Technology (NTNU), 7004 Trondheim, NO Norway; 2 NTNU Center for Health Promotion Research, Trondheim, Norway; 3 Department of Industrial Economics and Technology Management, Faculty of Economics and Management, NTNU, Trondheim, Norway

**Keywords:** Communication, Implementation, Hospital units, Health care personnel

## Abstract

**Background:**

To successfully achieve change in healthcare, a balance between technology and “people ware”, the human recourses, is necessary. However, the human aspect of the change implementation process has received less attention than the technological issues. The aim was﻿ to explore the factors that characterize the work culture in a hospital unit that successfully implemented change compared with the factors that characterize the work culture of a hospital unit with unsuccessful implementation.

**Method:**

The Systematizing Person-Group Relations method was used for gathering and analyzing data to explore what dominate the behavior in a particular work environment identifying challenges, limitations and opportunities. This method applied six different dimensions, each representing different behavior in a work culture: Synergy, Withdrawal, Opposition, Dependence, Control and Nurture. We compared two different units at the same hospital, one that successfully implemented change and one that was unsuccessful.

**Results:**

There were significant statistical differences between healthcare personnel working at a unit that successfully implemented change contrasted with the unit with unsuccessful implementation. These significant differences were found in both the synergy and control dimensions, which are important positive qualities in a work culture.

**Conclusion:**

The results of this study show that healthcare personnel at a unit with a successful implementation of change have a working environment with many positive qualities. This indicates that a work environment with a high focus on goal achievement and task orientation can handle the challenges of implementing changes.

## Background

To achieve successful changes in healthcare, a balance between technology and “people ware,” or human recourses, is essential. The human aspect of the change implementation process has received less attention than the technological issues [1]. These factors are important in developing a dynamic work culture that can cope with challenges, such as implementing of changes in of new procedures or technology.

The concept of implementation of change can be defined in many ways, in this article we use the definition presented by Richards and Hallberg [2]. Implementation is described as an “embedding of the new intervention into routine health care systems and activities”. They also highlighted that “implementation requires attention to multiple factors and is a highly active process” (p 13) that involves “use of strategies to adopt and integrate evidence-based health interventions and change practice patterns within specific settings” (p 13) [2].

Berg [3] posits that “successfully implementing patient care information systems in health care organizations appears to be a difficult task” and that “user-input must be a coherent steering force” (p 143). Another study identified educational campaigns, local adaption, and general agreement or guidelines for standard procedures as important tools for successful implementation [4]. To identify and describe barriers to implementation in healthcare, a structured decision-support procedure has been specified as crucial [5]. The quick progress and evaluation of technology-based interventions, without satisfactory understanding of or consideration of structural bottlenecks and other implementation barriers may influence a rise in the research–practice gap stated by Ramsey et al. [6]. Their study “emphasize the importance of developing technology-based tools that are more responsive to the needs and perspectives of behavioral health care providers to provide strategies to address major obstacles to the implementation and use of technology-based tools in health care” (p 68) [6]. Other researchers have also highlighted the importance of facilitating the implementation process for healthcare personnel. Lewy [7] notes that upcoming encounters for healthcare services will “use the knowledge in a way that will bring added value to healthcare professionals, healthcare organizations and patients without increasing workload” and “ develop solutions that can be easily integrated and used by healthcare professions considering the existing constraints” (p 2). The work environment and the climate in the work environment during implementation have been found to be crucial to success in another study they also recommended “managers should consider instituting specific organizational implementation policies and practices to increase positive perceptions of implementation climate” (p 1) [8].

A case study of a large-scale program undertaken in a hospital concluded that successful implementation was dependent on changes in work culture, relationships, and skills [9]. The concept of culture or organizational culture is not consistently defined in the literature. It can be thought of as the “normative glue” in organizations [1, 10, 11] or the sense making and control mechanisms that guide and shape the behavior and attitudes of the members of an organization. Both the concepts of organizational climate and organizational culture are used to illuminate the work culture in health care. For researchers and healthcare managers with responsibility for health service outcomes, there is a need to operationalize these concepts properly in order to measure them [12, 13]. Organizational culture has been defined as the norms, values and basic assumption shared by members of an organization [13, 14]. Organizational climate refers to members’ perception of organizational features such as decision- making, leadership and norms about the work [12, 13]. In this article, we will use the concept of work culture to describe both organizational culture and organizational climate.

Organizational processes are not as visible and measurable as technical issues and project management, and can often be regarded as “irrelevant topics such as feelings” [15]. If an organization is to change successfully, new and creative solutions must be developed to encourage the organization to renew itself [16]. When changes are introduced, resistance towards change may occur; in fact, one may find both resistance to and barriers against using new technology [17]. In handling and reducing this resistance, it is important to determine the content of these forms of resistance [18]. Challenges for introducing changes in health care units not only includes the behavior and intentions of the health care personnel, but also identifying motivational factors, such as focusing on the benefits of changes [1]. A positive attitude may influence an individual’s intention to change behavior [19].

Little is known about the issues in the work culture that may positively or negatively influence an implementation process. Acquiring this knowledge may contribute to making changes more sustainable in healthcare and bridging the rising research-practice gap. Based on this background we explored the following research question:

- What characterizes the work culture in a hospital unit that successfully implements change compared with one with an unsuccessful implementation?

## Method

The study was conducted to obtain knowledge about the work environment -with special focus on behavior and interaction between healthcare personnel- at two Hospital Medicine Units, one that successfully implemented change and one with unsuccessful implementation. This is a study conducted by St. Olavs Hospital, Trondheim University Hospital and Norwegian University of Science and Technology, Department of Nursing Science. This main aim of this study was to investigate the work culture at two different medicine units to see if the differences in the work culture can explain successful or unsuccessful implementation of change.

### Study design

This study was designed as a correlation study. One of the basic assumptions in this study are that predominant behaviors are an artifact of the typical work culture in the unit. We compared the results of two different units at the same hospital [20, 21]. The findings in the present study will be a comparison between the two units where successful and unsuccessful implementation had taken place. The units were chosen because the implementation were connected to the electronic patient record, and each unit had an introductory program related to the implementation. The implementation process was examined and characterized as either successful or unsuccessful [21–24]. Researchers who examined the sustainability of the implementation characterized units as either unsuccessful or successful in the implementation. In both cases, the implementations were regarding electronic patient records. In the study from 2004, a computer based tool for report assessment of symptoms and functioning were attempted to be implemented; in this case, the tool was not used at all [22, 23]. In the 2013 study, the use of nursing diagnoses in the electronic patient record were implemented [21, 24]. In both studies, using or not using the tool could be observed via the electronic patient record. During the implementation process in both units, a program with information, education and training was completed by all the health care personnel at the units [21–24]. In both cases, we followed the implementation while measuring the work culture at the two different units using the same questionnaire.

### Participants and data collection

In the spring of 2013, healthcare personnel working at the successful unit (succ) filled in and returned the questionnaire. Of the 101 healthcare workers working at the unit, 70 participated (69%). The sample of healthcare personnel consisted of 63 nurses, 6 assistant nurses, and 2 nursing managers; there were 69 females and 2 males. The questionnaires were distributed and filled in at seminars about the implementation. Only healthcare personnel working more than half time were invited to participate in the study. These findings were compared with findings from an earlier study conducted in 2004. Of the 36 healthcare personnel working at the unsuccessful unit (unsucc), 25 (70%) filled in and returned a questionnaire. The sample consisted of 17 nurses, 2 physicians, 2 physiotherapists, and 4 other professions. There were 24 females and 1 male in the sample. The questionnaires were distributed and filled in at morning meetings (taking approximately 10 min and with researcher present to answer questions) or delivered to the mailboxes of those who were not present at the meeting. Two follow- ups were conducted.

The sociodemographic data were equal for the personnel who participated and those who did not. The personnel who worked night shifts were more likely to not participate in the study.

In total, this present study consisted of 106 healthcare personnel. The university hospital involved in the study had 993 beds and 59,016 hospitalizations in 2013.

### The instrument and data analysis

The Systematizing Person-Group Relations Instrument (SPGR) was used for data gathering and investigation [14, 25–27]. The SPGR process is based on the “Semantic Differential scaling technique” established by Osgood [28]. Earlier studies [29–31] have described the validity and reliability of the SPGR tool and the instrument has been used in different settings [32–34]. The subsequent detailed appearance of the SPGR tool is presented similarly to the methodological descriptions in an earlier study [32].

The SPGR scale consists of 24 items describing organizational behavior. Each item is rated on a scale of the behavior described as occurring never or seldom (1), sometimes (2) or often (3). The organizational behaviors are described along dimensions labeled as; Control versus Nurture (C-N), Opposition versus Dependence (O-D), and Withdrawal versus Synergy (W-S), where the value for the poles of the three dimensions’ results from the ratings of four of the 24 items. A brief description of the behavior describing each dimension is given in Table 1.

**Table 1:** Elements of group constitution based on SPGR instrument

Dimension	Group function	Short description
C-N	Control	Structure, logic, authority
	Nurture	Caring, social orientation, openness
O-D	Opposition	Criticism, rebellion
	Dependence	Loyalty, conformance, submission
W-S	Withdrawal	Passive resistance
	Synergy	Engagement, constructive goal-oriented teamwork

**Fig. 1:**
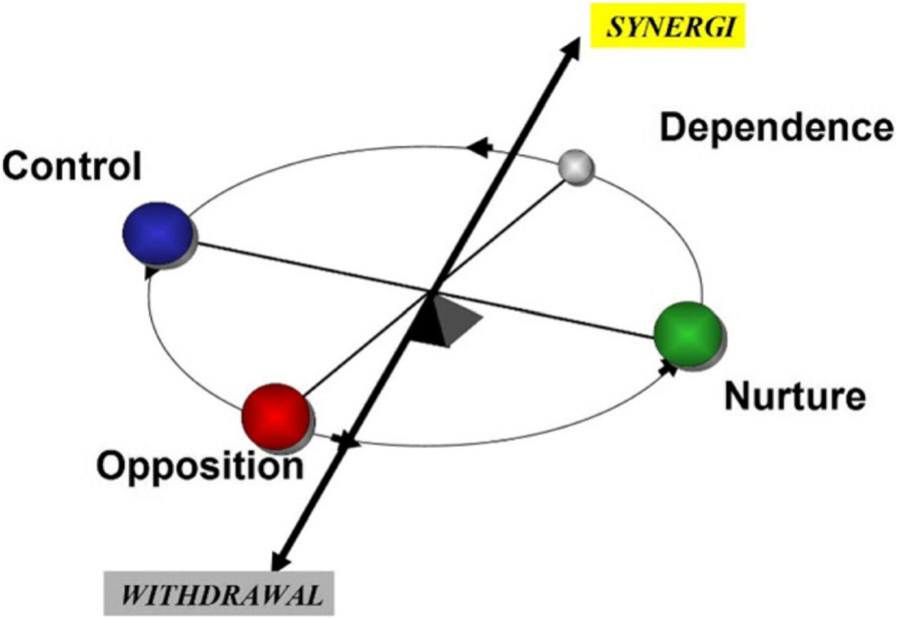
The SPGR instrument relies on a factor analytical model consisting of these basic dimensions

The “Control” pole of the C-N dimension includes analytical, task-oriented, or autocratic behavior and the “Nurture” pole includes caring, empathic, or spontaneous behavior. Along the O-D dimension, “Opposition” includes critical, assertive, or self-sufficient behavior and “Dependence” is passive and obedient behavior. Along the S-W dimension, “Synergy” includes engagement and constructive goal-orientated behavior, and “Withdrawal” is passive rebellion and resignation [14, 27, 32]. The theoretical foundation for SPGR and psychometrics has been elaborated in the work of Sjøvold [14, 27]. A further discussion of the psychometrics of the SPGR methodology can be found in the SPGR manual [31]. In Table 2, the results of the survey are presented by two facets for each pole of the SPGR dimensions (eg C1 and C2 for C) and transposed to a nine-point scale (Fig. 1).

**Table 2:** Work culture within units

Vector	Code	Typical behavior	succ	unsucc	
Ruling	C1	Controlling, autocratic, attentive to rules and procedures	4.87	2.52	**
Task-orientation	C2	Analytical, task-oriented, conforming	6.37	5.04	*
Caring	N1	Taking care of others, attentive to relations	8.09	5.58	**
Creativity	N2	Creative, spontaneous	1.97	2.61	
Criticism	O1	Critical, opposing	3.02	2.07	*
Assertiveness	O2	Assertive, self-sufficient	3.37	2.70	
Loyalty	D1	Obedient, conforming	7.67	4.59	**
Acceptance	D2	Passive, accepting	7.89	6.48	**
Resignation	W1	Sad appearance, showing lack of self-confidence	1.56	2.34	*
Self-sacrifice	W2	Passive, reluctant to contribute	1.85	2.52	
Engagement	S1	Engaged, inviting others to contribute	8.12	6.48	**
Empathy	S2	Showing empathy and interest in others	7.86	6.75	*

*p < 0.05 ** *p* < 0.01

### Statistical analysis

Independent samples of students’ t-tests were conducted to look for differences between the two selected perspectives based on the findings and the two samples were correlated. The relevant data was analyzed using the Statistical Package for Social Sciences (SPSS Inc., Chicago, IL, USA) version 21.0 for Windows. All research hypotheses were tested at the 0.05 and 0.01 significance level for the two-tailed test. Checking for normality gave acceptable results in our sample for the test used.

### Ethical considerations

The ethical guidelines of voluntary participation and the possibility of withdrawal at any point were followed. All gathered data was anonymized. The Regional Committees for Medical and Health Research Ethics in Norway assessed the study as a quality assessment project of the actual hospital. Based on that the actual department management and the privacy ombudsman at the hospital sanctioned the study.

## Results

The health care personnel were asked about issues in their work culture within the unit, and we compared the results from these two units as seen in Table 2. There were significant statistical differences in 9 of the 12 vectors. Five of the vectors have significant differences of a *p* < 0.01 level, while 4 at a *p* < 0.05 level.

The results revealed that the health care personnel working at the successful (succ) unit described their working culture differently than the healthcare personnel at the unsuccessful unit (unsucc). At the succ unit participants described their work environment as characterized by high values on the vectors ruling (C1, mean 4.87 vs. unsucc 2.52), task-orientation (C2, mean 6.32 vs unsucc 5.04), caring (N1 mean 8.09 vs unsucc 5.58), criticism (O1, mean 3.02 vs unsucc2.07), loyalty (D1, mean 7.67 vs unsucc 4.59), acceptance (D2, mean 7.89 vs unsucc 6.48), engagement (S1, mean 8.12 vs unsucc 6.48) and empathy (S2, mean 7.86 vs unsucc 6.75). Furthermore, resignation (W1, mean 1.56 vs unsucc 2.34) had lower scores than the scores at the unsucc unit. Creativity falls in the “Nurture” dimension, whereas resignation and self-sacrifice are both in the “Withdrawal” dimension, and criticism is in the “Opposition” dimension. The empathy and engagement vectors belong to the “Synergy” dimension, and acceptance and loyalty belong to the “Dependence” dimension, whereas caring is in the “Nurture” dimension and task-orientation and ruling are in the “Control” dimension (see Table 2).

## Discussion

To find the factors that characterize the behavior in a healthcare unit with successful implementation, we compared the results with those from an unsuccessful implementation. We wanted to explore research questions about what characterizes the work culture in a hospital unit with successful implementation compared with one with an unsuccessful implementation.

### What are the difference between the two units

It seems like the work environment at succ unit are characterized by high influences in ruling (C1), task-orientation (C2), caring (N1), criticism (O1), loyalty (D1), acceptance (D2), engagement (S1), empathy (S2) and less by resignation (W1). Task-orientation (C2), caring (N1), engagement (S1), and empathy (S2) can be characterized as positive qualities in the work culture, so long as they do not contribute to imbalance related to the other vectors. Both empathy and engagement belong to the “Synergy” dimension, which is important in organizations for developing a higher level of maturity in both independent work and collaboration [26]. However, resignation (W1) represents negative qualities in the work culture; findings reported in Table 2 indicate that the respondents at the unsucc unit experience higher levels of self-sacrificing and resignation than the succ group. The high mean scores on the self-sacrificing vector, may be associated with employees who are not always doing their tasks with joy, and can yield fertile conditions for a culture of complaining, dissatisfaction, and passivity. Both the vectors ruling (C1) and task orientation (C2) can be positive qualities in a changing process because the properties of the vectors can develop into a more efficient way of dealing with the changes, and can also contributed to a feeling of autonomy and having decision-making opportunities in the work culture [35]. The vectors loyalty (D1) and acceptance (D2) can also further the implementation process. When healthcare workers feel loyal and accepting of changes, they accept the task of working towards a common goal. Also, when making changes and implementing them it is important to be critical in a constructive way. The high scores in task-orientation (C2), caring (N1), and engagement (S1) indicate that this is present in the succ unit. This can lead to both collaborative relationships and promote decision making [36].

Task-orientation can be viewed as a more “high tech” approach to the patients’ situation, especially if this includes implementation of new technology [1, 22]. Earlier findings show that computer technology influenced the communication between the patient and healthcare personnel and lead to an “artificial way of communication” [22].

To facilitate changes in healthcare, it is important to influence the behavior and intentions of the healthcare personnel [1]. Both behavior intentions and behavior are influenced by several factors such as attitudes, norms, and motivation, and are well-described [19, 37]. Influencing values and norms is generally difficult, whereas motivation and attitudes are more susceptible to influence and may be influenced by the healthcare personnel’s present life situation [1].

### How may these differences affect the implementation process?

As found in another study [3] user input is important to a successful implementation process. In this study, we found that the respondents at the succ unit had higher scores on the vectors task-orientation (C2) and engagement (S1), which may indicate a work culture that promotes discussions where the health care personnel may express their opinions. Together with high scores on the vectors loyalty (D1) and acceptance (D2), these attributes can promote general agreement, which has been found to be an important factor in obtaining successful implementation [4]. Furthermore, the high scores on the vectors task-orientation (C2), loyalty (D1), and acceptance (D2) from the respondents shows a work culture at the succ unit that could point toward the use of structured decision support procedures in the implementation process at this unit, as found to be crucial in another study [5]. The rising research - practice gap described by Ramsey [6], may be bridged by supporting both task-oriented and engagement behavior among healthcare personnel when changes are introduced and implementation is begun. This may also contribute to a more positive implementation climate and a focus on the existing constraints in the work culture, described by other studies as a manager’s responsibility to foster [7, 8].

### Limitations of the present study

This study also has limitations. The two units had different implementation programs, but both units participated in an implementation program before the implementations. Many years passed between the two studies in this article, and other aspects may have an influence on the work culture. However, the present findings can give an indication as to the direction that research ought to follow in subsequent studies. In subsequent studies, a control group could be used to see if the results differ in a unit without an implementation process. The response rate in both units were high with 69% and 70% participation respectively. Nevertheless, the sampling process may have influenced the results if only the most enthusiastic healthcare personnel participated; and we have little knowledge about the non-respondents. This study was conducted in Norway with a Norwegian population. In Norway, work conditions are usually favorable for workers, so the results of this research could not be generalizable to other contexts without taking that into consideration [38]. Furthermore, the study has been carried out in a field where this focus has not been thoroughly described previously.

## Conclusion

The results of this study shows that healthcare personnel at the successful unit had a work culture with many positive qualities, including a good balance between independence, engagement, loyalty and acceptance. Furthermore, a work culture with a high focus on goal achievement and task-orientation, as the successful unit had, may handle the challenges inherent in implementation of changes in a better way. User input, autonomy, and engagement found in other studies to be important, were also found in the work culture at the successful unit in this study. High levels of empathy are also vital factors in an implementation process and can influence the work culture by creating a higher level of maturity among the healthcare personnel both in independent work and in collaboration.

## Abbreviations

C: Control
C1: Ruling
C2: Task-orientation
D: Dependence
D1: Loyalty
D2: Acceptance
N: Nurture
N1: Caring
N2: Creativity
O: Opposition
O1: Criticism
O2: Assertiveness
S: Synergy
S1: Engagement
S2: Empathy
SPGR: The Systematizing Person-Group Relations Instrument
SPSS: Statistical Package for Social Sciences
Succ: Successful unit
Unsucc: Unsuccessful unit
W: Withdrawal
W1: Resignation
W2: Self-sacrifice

## References

[1] Andre B, Ringdal GI, Loge JH, Rannestad T, Laerum H, Kaasa S (2008). Experiences with the implementation of computerized tools in health care units: a review article. Int J Hum Comput Interact.

[2] Richards D, Hallberg IR (2015). Complex interventions in health an overview of research methods.

[3] Berg M (2001). Implementing information systems in health care organizations: myths and challenges. Int J Med Inf.

[4] Sijpkens MK, Steegers EA, Rosman AN (2016). Facilitators and barriers for successful implementation of interconception care in preventive child health care services in the Netherlands. Matern Child Health J.

[5] Craig LE, Churilov L, Olenko L, Cadilhac DA, Grimley R, Dale S, Martinez-Garduno C, McInnes E, Considine J, Grimshaw JM (2017). Testing a systematic approach to identify and prioritise barriers to successful implementation of a complex healthcare intervention. BMC Med Res Methodol.

[6] Ramsey A, Lord S, Torrey J, Marsch L, Lardiere M (2016). Paving the way to successful implementation: identifying key barriers to use of technology-based therapeutic tools for behavioral health care. J Behav Health Serv Res.

[7] Lewy H (2015). Wearable technologies–future challenges for implementation in healthcare services. Healthc Technol Lett.

[8] Jacobs SR, Weiner BJ, Reeve BB, Hofmann DA, Christian M, Weinberger M (2015). Determining the predictors of innovation implementation in healthcare: a quantitative analysis of implementation effectiveness. BMC Health Serv Res.

[9] Bate P (2000). Changing the culture of a hospital: from hierarchy to networked community. Public Adm.

[10] Sleutel MR (2000). Climate, culture, context, or work environment? Organizational factors that influence nursing practice. JNursAdm.

[11] Boëthius SB, Ögren M-L, Sjøvold E, Sundin EC (2004). Experiences of group culture and patterns of interaction in psychotherapy supervision groups. Clin Superv.

[12] Stone P, Harrison MI, Feldman P, Linzer M, Peng T, Roblin D, Scott-Cawiezell J, Warren N, Williams ES (2005). Organizational climate of staff working conditions and safety—an integrative model. Adv Patient Safety.

[13] Gershon RRM, Stone PW, Bakken S, Larson E (2004). Measurement of organizational culture and climate in healthcare. J Nurs Adm.

[14] Sjøvold E. Systematizing Person-Group Relations (SPGR) - A Field Theory of Social. Small Group Res. 2007;38(5):615–35.

[15] Lorenzi NM (2004). Beyond the gadgets - non-technological barriers to information systems need to be overcome too. Br Med J.

[16] Cummings TG, Worley CG (2001). Organization Development & Change.

[17] Lorenzi NM, Riley RT (2000). Managing change: an overview. JAmMedInformAssoc.

[18] Lorenzi NM, Riley RT, Dewan NA (2001). Barriers and resistance to informatics in behavioral health. Medinfo.

[19] Strobe W (2008). Social psychology and health.

[20] Andre B, Sjovold E, Rannestad T, Holmemo M, Ringdal GI. Work culture among healthcare personnel in a palliative medicine unit. Palliative & supportive care. 2012:1–6. 10.1017/S1478951512000818 23089522

[21] Frigstad SA, Nøst TH, André B (2015). implementation of free text format nursing diagnoses at a university Hospital’s medical department**.** Exploring Nurses’ and Nursing Students’ Experiences on Use and Usefulness A Qualitative Study. Nurs Res Pract.

[22] Andre B, Ringdal GI, Loge JH, Rannestad T, Kaasa S (2009). Implementation of computerized technology in a palliative care unit. Palliative & supportive care.

[23] Andre B, Ringdal GI, Loge JH, Rannestad T, Kaasa S (2008). The importance of key personnel and active management for successful implementation of computer-based technology in palliative care: results from a qualitative study. Computers, informatics, nursing : CIN.

[24] Nøst TH, Frigstad, SA. André, B. : Impact of an Educational Intervention on Nursing Diagnoses in free-text format in Electronic Health Records. Nord J Nurs Res 2016, In press**.**

[25] Hare AP, Sjøvold E, Baker HG (2005). Analysis of social interaction systems: SYMLOG research and applications: University Press of America.

[26] Sjøvold E (2006). Maturity and effectiveness in small groups. Nordic Psychology.

[27] Sjøvold E, Hare A, Sjovold E, Hare AP, Sjovold E, Baker & Powers (2005). Bions theory on group emotionality. Analysis of social interaction systems.

[28] Osgood CE (1957). The measurement of meaning: University of Illinois press.

[29] Koenigs RJ, Hare SE, Hare AP (2002). SYMLOG reliability and validity.

[30] Koenigs RJ, Hare SE, Hare AP, Cohen MA, Hare E, Sj›vold HG, Baker J (2005). reliability and validity In A.P. Powers, Analysis of Social Systems.

[31] Sjøvold E. The SPGR manual. Oslo: SPGR publishing; 2002.

[32] André B, Frigstad SA, Nøst TH, Sjøvold E: Exploring nursing staffs communication in stressful and non-stressful situations. J Nurs Manag 2015:n/a-n/a. 10.1111/jonm.12319 26077500

[33] Heldal F, Sj›vold E, Heldal AF (2004). success on the internet-optimizing relationships through the corporate site. Int J Inf Manag.

[34] Schultz JS, Sjøvold E, André B (2017). Can work climate explain innovative readiness for change?. The Journal of Organizational Change Management.

[35] Erenstein CF, McCaffrey R (2007). How healthcare work environments influence nurse retention. Holist Nurs Pract.

[36] Heath J, Johanson W, Blake N (2004). Healthy work environments: a validation of the literature. J Nurs Adm.

[37] Ajzen I (1991). The theory of planned behavior. Organ Behav Hum Decis Process.

[38] Andre B, Sjøvold E, Holmemo M, Rannestad T, Ringdal GI (2013). Expectations and desires of palliative health care personnel concerning their future work culture. J Hosp Adm.

